# Witnessing a possible revolution in antiplatelet therapy for coronary artery bypass grafting

**DOI:** 10.1093/ehjcvp/pvaf068

**Published:** 2025-09-12

**Authors:** Mattia Galli, Felice Gragnano, Mario Gaudino

**Affiliations:** Department of Medical-Surgical Sciences and Biotechnologies, Sapienza University of Rome, Corso della Repubblica, 79, Latina 04100, Italy; Maria Cecilia Hospital, GVM Care & Research, Cotignola 48033, Italy; Department of Translational Medical Sciences, University of Campania ‘Luigi Vanvitelli’, Caserta, Italy; Division of Clinical Cardiology, A.O.R.N. ‘Sant’Anna e San Sebastiano’, Caserta, Italy; Department of Cardiothoracic Surgery, Weill Cornell Medicine, 525 East 68th Street, New York 10065, USA

Current guidelines recommend 12 months of dual antiplatelet therapy (DAPT) with a new-generation P2Y_12_ inhibitor (ticagrelor or prasugrel) in patients with acute coronary syndrome (ACS), including those undergoing coronary artery bypass grafting (CABG).^1^ However, in the CABG setting, this recommendation is based only on expert opinion (level of evidence C), supported primarily by observational studies and small or *post hoc* analyses of randomized controlled trials (RCTs).^1^

This year, the European Society of Cardiology Congress featured the presentation of two pivotal RCTs in this setting: *Dual Antiplatelet Therapy with Ticagrelor and Acetylsalicylic Acid vs. ASA Only after Isolated Coronary Artery Bypass Grafting in Patients with Acute Coronary Syndrome* (TACSI) and *Timing of Platelet Inhibition after Coronary Artery Bypass Grafting* (TOP-CABG). In the TACSI trial, one year of ticagrelor plus aspirin did not reduce the incidence of death, myocardial infarction, stroke, or repeat revascularization compared with aspirin alone in 2201 patients from north Europe who underwent CABG for ACS within the prior 6 weeks. Notably, Kaplan–Meier curves seemed to favour DAPT during the first 3 months, suggesting that a short initial course of DAPT may still be beneficial.^2^ To this extent, the TOP-CABG trial (NCT05380063), which randomized 2290 elective CABG patients from China to 12 months of aspirin plus ticagrelor or 3 months of DAPT followed by 9 months of aspirin alone, found the shorter regimen to be non-inferior for the primary endpoint of graft occlusion at 1 year.

Collectively, these trials support a paradigm shift towards de-escalating antiplatelet therapy after CABG, suggesting that 12-month DAPT may be outdated. However, key uncertainties remain regarding long-term efficacy, the clinical impact of graft occlusion, the optimal DAPT duration in elective vs. urgent CABG, and whether P2Y_12_ inhibitor monotherapy may outperform aspirin or is influenced by factors such as ethnicity or sex.^3–5^ Ongoing RCTs, including ODIN (NCT05997693) and SDAT-IRC (NCT03789916), are expected to provide further insights.

## Data Availability

No new data were generated or analysed in this manuscript.

## References

[1] Sandner S, Gaudino M, Agewall S, Bonalumi G, Bonaros N, Czerny M, Jeppsson A, Milojevic M, Niessner A, Parolari A, Sulzgruber P, Tamargo J, Thielmann M, Wassmann S, Zientara A, Dobrev D. Antithrombotic therapy after coronary artery bypass graft surgery: a clinical consensus statement of the ESC Working Group on Cardiovascular Surgery, the ESC Working Group on Cardiovascular Pharmacotherapy, and the European Association for Cardio-Thoracic Surgery (EACTS). Eur Heart J 2025:ehaf423. 10.1093/eurheartj/ehaf423. 40884067

[2] Jeppsson A, James S, Moller CH, Malm CJ, Dalén M, Vanky F, Modrau IS, Andersen K, Anttila V, Atroshchenko GV, Barbu M, Dreifaldt M, El-Akkawi AI, Friberg Ö, Gudbjartsson T, Gunn J, Haaverstad R, Halonen J, Hansson EC, Holm J, Husso A, Juvonen T, Jakobsen Ø, Jideus L, Johannesson E, Jonsson Holmdahl A, Jonsson K, Kolseth SM, Krasniqi L, Mäkelä T, Mennander A, Mohagen Krogstad LE, Rafiq S, Raivio P, Riber L, Tahir A, Thorsen C, Tønnessen T, Wahba A, Zindovic I, Pivodic A, Nielsen SJ, Erlinge D, Alfredsson J, Sartipy U; TACSI Trial Group. Ticagrelor and aspirin or aspirin alone after coronary surgery for acute coronary syndrome. N Engl J Med. 2025. 10.1056/NEJMoa2508026. 40888737

[3] Galli M, Laudani C, Occhipinti G, Spagnolo M, Gragnano F, D'Amario D, Navarese EP, Mehran R, Valgimigli M, Capodanno D, Angiolillo DJ. P2y12 inhibitor monotherapy after short DAPT in acute coronary syndrome: a systematic review and meta-analysis. Eur Heart J Cardiovasc Pharmacother 2024;10:588–598. 39054275 10.1093/ehjcvp/pvae057

[4] Galli M, Laborante R, Occhipinti G, Zito A, Spadafora L, Biondi-Zoccai G, Nerla R, Castriota F, D'Amario D, Capodanno D, Jeong Y-H, Kimura T, Mehran R, Angiolillo DJ. Impact of ethnicity on antiplatelet treatment regimens for bleeding reduction in acute coronary syndromes: a systematic review and pre-specified subgroup meta-analysis. Eur Heart J Cardiovasc Pharmacother 2024;10:158–169. 37960983 10.1093/ehjcvp/pvad085

[5] Galli M, Terracina S, Schiera E, De Corci S, Sangiorgi D, Mancone M, Frati L, Sciarretta S, Angiolillo DJ, Pulcinelli FM. Sex-related variations in platelet reactivity in presence or absence of antiplatelet therapy. Eur Heart J Cardiovasc Pharmacother 2025;11:509–517. 40366913 10.1093/ehjcvp/pvaf034 PMC12450592

